# Wearables and Atrial Fibrillation: Advances in Detection, Clinical Impact, Ethical Concerns, and Future Perspectives

**DOI:** 10.7759/cureus.77404

**Published:** 2025-01-13

**Authors:** Antonino Francisco, Capela Pascoal, Pedro Lamborne, Humberto Morais, Mauer Gonçalves

**Affiliations:** 1 Medical School, Centro de Estudos Avançados em Educação e Formação Médica (CEDUMED) Faculdade de Medicina, Universidade Agostinho Neto, Luanda, AGO; 2 Cardiology, Centro de Estudos Avançados em Educação e Formação Médica (CEDUMED) Faculdade de Medicina, Universidade Agostinho Neto, Luanda, AGO; 3 Cardiology, Hospital Militar Principal/Instituto Superior, Luanda, AGO; 4 Cardiology, Luanda Medical Center, Luanda, AGO

**Keywords:** artificial intelligence in medicine, atrial fibrillation (af), atrial fibrillation management, smart watches, wearables

## Abstract

Atrial fibrillation (AF), the most common cardiac arrhythmia, is associated with a significantly increased risk of stroke, heart failure, and mortality. Early diagnosis and management are crucial to mitigating these risks. Wearable devices such as smartwatches and fitness bands, enhanced by advanced artificial intelligence (AI) algorithms, offer a promising solution for early AF detection due to their accessibility, ease of use, and cost-effectiveness. Although the ability of these algorithms to identify AF has been authorized, critical questions remain about their integration into clinical practice, ethical implications, and long-term benefits. This review uniquely explores the intersection of wearable technology and AF management, providing a detailed analysis of current evidence, emerging trends, and the challenges associated with these innovations.

## Introduction and background

Atrial fibrillation (AF) is a condition in which the electrical impulses in the atria become rapid and disorganized, overriding the heart's natural pacemaker, irregular baseline made up of "f" (fibrillation) waves discharging at a frequency of 350 to 600 beats/min. These electrical impulses are triggered from multiple areas within and around the atria, rather than from a single area like the sinus node. This disrupts proper heart rate control, resulting in irregular heartbeats [1,2].

The growing prevalence of AF is largely a reflection of increasing global life expectancy, which contributes to the aging of populations worldwide. Findings from the Framingham Heart Study demonstrated a fourfold increase in the age-adjusted prevalence of AF over a 50-year follow-up, underscoring the significant impact of this condition in aging populations [3]. 

Globally, it is estimated that AF affected approximately 60 million people in 2019, and this number is expected to rise exponentially [4]. In the United States alone, projections suggest that the number of individuals diagnosed with AF could surpass 12.1 million by 2030 [5]. This growth reflects a combination of factors, including longer life expectancy, aging demographics, and improvements in AF detection.

The prevalence and incidence of AF increase significantly with age and are more common in men than in women. This difference is partially attributed to sex-specific variations in risk factors for AF. However, the lifetime risk for AF development appears to be nearly equivalent between men and women in North American and European populations. This equivalence is likely due to women’s longer life expectancy, allowing them to reach the cumulative incidence of AF observed in men at later ages [6].

AF is associated with high rates of morbidity and mortality, increasing the risk of stroke by five times, as well as the risk of heart failure, and it can also cause a variety of symptoms and negatively affect the quality of life [7]. The clinical presentation of atrial fibrillation can be quite heterogeneous among patients, ranging from asymptomatic cases to death. The episodic nature of clinical manifestations and the often nonspecific symptoms make the diagnosis and management of these patients challenging [8]. Subclinical atrial fibrillation accounts for approximately one-third of the total population with AF and is also associated with an increased risk of stroke. Asymptomatic AF carries a similar risk of all-cause mortality, cardiovascular mortality, and stroke/thromboembolism compared to symptomatic AF [9].

Smartwatches and fitness bands can passively measure heart rate from the wrist using photoplethysmography (PPG) from an optical sensor. Longitudinal pulse data can be analyzed in real-time to assess pulse irregularity and variability, thus emerging as a promising tool for early AF detection [10]. In selected asymptomatic patients, early detection of atrial fibrillation may be crucial in preventing stroke and heart failure, through strategies such as oral anticoagulation and control of rhythm and/or heart rate [11,12].

**Figure 1:**
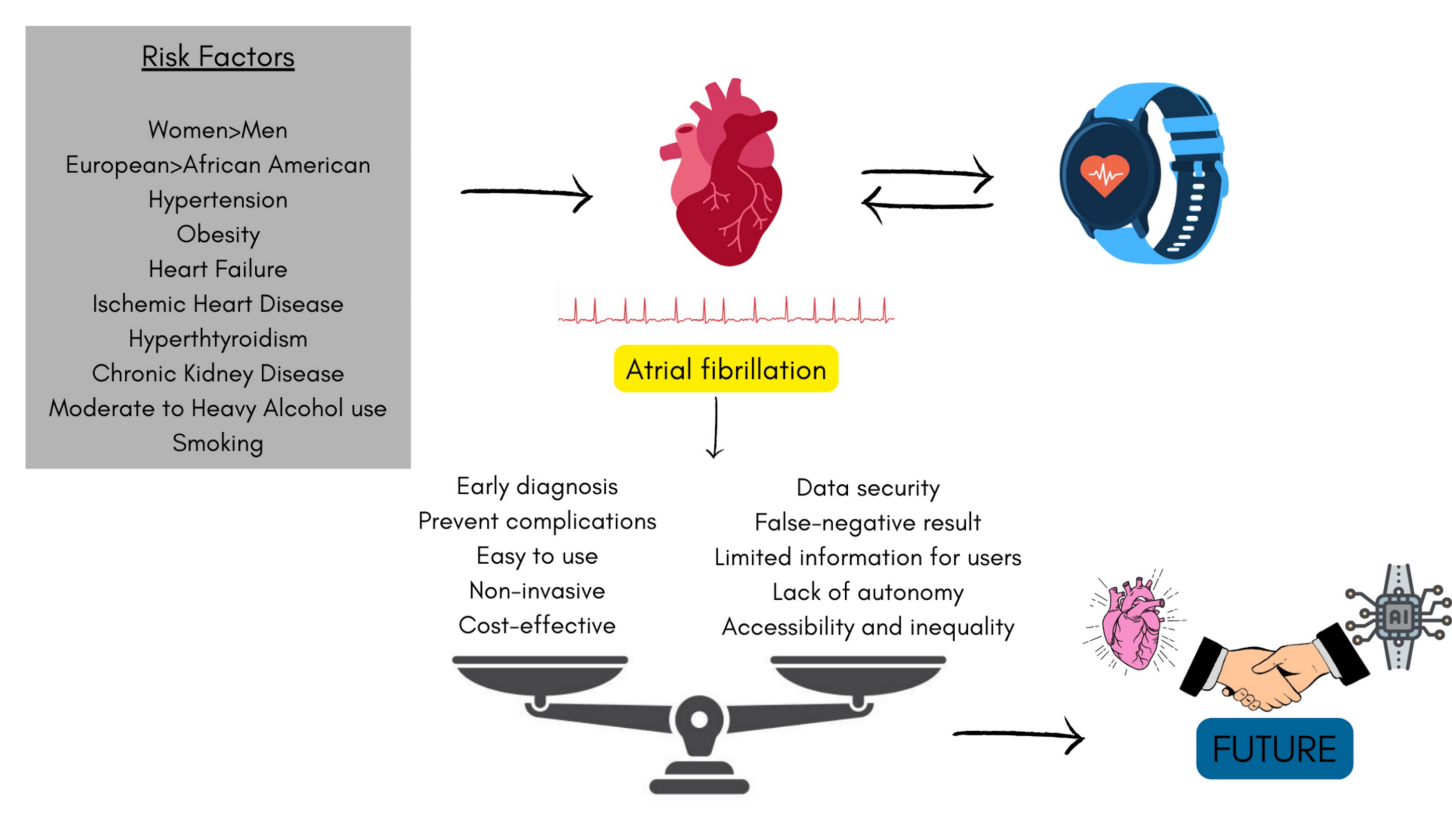
Wearables in the diagnosis of atrial fibrillation. The image has been created by the authors.

A sequence of events is illustrated in the graphical summary below, starting with the influence of cardiovascular risk factors on the development of atrial fibrillation, the use of wearables in the detection of atrial fibrillation, and the advantages and disadvantages of these devices. Understanding and balancing these factors will help identify groups of patients who could benefit from these devices in the future (Figure 1).

Despite recent studies highlighting the potential benefits of these devices in the early diagnosis of AF and the management of these patients, these devices or algorithms may not be risk-free, and their improper use can cause harm [13-15]. The use of electrocardiogram (ECG) is still required to confirm the diagnosis of AF, and despite growing evidence, the inclusion of smartwatches in the diagnosis and management of patients with AF has not yet been established [16].

This critical narrative review aims to present the current state of the art regarding the use of wearable devices in patients with a higher risk for atrial fibrillation. As AF continues to rise globally, driven by aging populations and evolving technology, the integration of smartwatches and wearable technologies into clinical practice could revolutionize early detection and management.

## Review

Atrial fibrillation

Given the growing prevalence and impact of AF in an aging population, prioritizing strategies for prevention, early detection, and effective management is critical to mitigating the global burden of this condition, which is poised to become a significant public health challenge in the coming decades.

Recent research has identified that a lack of awareness about AF results in a highly unfavorable prognosis, including a significantly elevated mortality risk of up to 94% [17]. To increase awareness of AF, ensure early detection, improve access to treatment, and enhance outcomes, ambulatory monitoring devices technologies such as the Holter monitor, ECG patches, and implantable loop recorders have been developed, providing highly accurate diagnosis accuracy with sensitivity and specificity of at least 93-96% [18-20].

With early diagnosis of AF, it becomes possible to significantly reduce complications, such as cardioembolic stroke and its recurrence, through appropriate monitoring and early use of anticoagulants [21-24]. The early detection of atrial fibrillation faces significant limitations when using Holter monitoring [18,25]. One of the main challenges is the short recording duration, which typically ranges from 24 to 48 hours, often insufficient to identify episodes of AF in many patients. Although devices like ECG patches allow monitoring for up to 14 days [26], their effectiveness remains uncertain, as some studies report that in cases of cryptogenic stroke, the average time to detect AF can reach 30 days. On the other hand, implantable loop recorders provide a solution for long-term monitoring. However, this approach is not widely adopted, as the procedure is invasive and the high cost discourages many patients from choosing this technology [27,28].

In this context, new modalities have emerged, designed to increase adherence, facilitate convenience, and reduce financial healthcare costs without compromising quality of care. Specifically, smartphones and smartwatches can enhance AF detection in the subclinical phase [29]. Since stroke is the first manifestation of AF in around 25% of cases, early detection of this condition can help reduce its impact as the leading cause of disability [30].

Types of sensors

Understanding the different technologies is crucial to gaining a clearer insight into their clinical application, as well as the potential errors, limitations, and challenges involved [31]. Most consumer-focused arrhythmia monitoring devices use photoplethysmography (PPG), a technology that evaluates volumetric variations in blood flow based on the intensity of light reflected on the skin. The generated signal shows peaks corresponding to pulsatile blood flow, and the interval between these peaks is proportional to the R-R interval. Due to its simplicity in detecting irregularities, PPG technology has been primarily validated for identifying AF [32,33]. However, factors such as skin tone, skin moisture, and the presence of tattoos can interfere with the results. For example, a dark tattoo in the area where the sensor is placed may interfere with signal readings, potentially causing inconsistencies that could lead to a false detection of AF. Similarly, a patient with extensive tattoos on the wrist may have undetected AF, as the sensor may not be able to generate an adequate signal for analysis [34,35].

An alternative approach to monitoring heart rate and rhythm involves the use of a single-lead electrocardiogram (ECG), where specific parts of a watch act as positive and negative electrodes. While this technology is useful in certain situations, its ability to detect and analyze more complex heart rhythms is limited [36]. Furthermore, portable devices on the market vary widely in terms of the sensors used, ranging from smart fabrics, also known as e-textiles, and clothing equipped with sensors, to more sophisticated devices. Examples include ECG sensors, such as those integrated into Apple Watches (Apple Inc., Cupertino, CA, USA); near-field communication (NFC) sensors, common in smartphones; and sensors based on PPG, found in devices like pulse oximeters. Each type of sensor has specific advantages and limitations, influencing its clinical applicability and effectiveness in different scenarios [37]. In comparison between PPG and ECG-based technologies, the results regarding the superiority of one over the other were mixed [31,33]. Finally, smartphone applications, despite requiring greater participation and effort on the part of the user compared to passive detection, showed extremely high sensitivity and specificity of 97.0% and 93.5%respectively [38].

Current evidence

Several studies have reported the use of wearable technologies. In the Apple Heart study, with more than 400,000 participants with no previous diagnosis of AF, those with an irregular pulse were seen by telemedicine and given an ECG monitor. Among 2,064 notified patients, the positive predictive value for detection was 84% [39].

A study that evaluated the effect of AF detection using a Zio patch (iRhythm Technologies, Inc., San Francisco, CA, USA) showed a 3.0% increase in AF detection, (with a 95% confidence interval of 1.8-4.1) over a period of four months [40]. Another study validated the use of deep neural networks to detect AF from Apple Watch data, involving two distinct cohorts: one consisting of patients undergoing cardioversion and the other of outpatients with self-reported AF. Using ECG as a reference for comparison, sensitivity reached 98% and specificity was 90.2% in the first group, presenting results similar to those found in a previous study, which obtained 93% sensitivity and 84% specificity in patients undergoing cardioversion as well [41]. However, in the second group, which is closer to the practical setting of technology use, the sensitivity and specificity values were considerably lower, with sensitivity at 67.7% and specificity at 67.6% [42]. These data highlight the differences in the algorithm’s performance under controlled conditions and in clinical practice, suggesting that the use of technologies such as the Apple Watch for AF monitoring can vary significantly depending on the context in which they are applied [42].

Two studies revealed that AF detection by smartphones has high specificity (94%) and sensitivity (96%), and also demonstrated that smartwatches are not inferior to medical-grade devices for this purpose [43,44]. In one study, participants were monitored three times a day, and whenever they felt palpitations, the results showed that the KardiaMobile (AliveCor, Inc., Mountain View, CA, USA) device detected AF more accurately than 24-hour ECGs (9.4% versus 2%) [45]. In another study, more than 1,000 patients with no history of AF were randomly divided between standard devices and twice-weekly monitoring with the KardiaMobile. The results showed that the AF detection rate was 3.8% in the group using the KardiaMobile, compared to less than 1% in the group receiving the standard device [46]. Due to the need to remove them for charging, smartwatches are not worn continuously, which reduces their sensitivity for detecting infrequent paroxysmal arrhythmias [32].

An observational study, conducted to evaluate the performance and usability of a smartwatch for heart rhythm analysis in elderly individuals with a mean age of 71 years, who had AF or risk factors for the condition, compared to the gold standard, revealed that the device’s algorithm performed exceptionally well, with a sensitivity of 98.2%, specificity of 98.1%, and accuracy of 98.1% in identifying irregular pulses consistent with atrial fibrillation. Despite challenges such as advanced age, lack of familiarity with smartwatch technology, and a high burden of comorbidities, participants reported a high level of acceptance of the smartwatch, demonstrating that even under challenging conditions, patients are able to use the device effectively for monitoring and follow-up of their clinical condition [47].

The SAFE (Screening for Atrial Fibrillation in the Elderly) study highlighted the economic viability of comprehensive screening for AF in individuals aged 65 and older [48]. The findings underscored that such targeted screening not only facilitates a significant increase in the detection of AF cases but also contributes to a meaningful reduction in the incidence of ischemic strokes, demonstrating its cost-effectiveness [48]. Consistent with these findings, current clinical guidelines for AF management recommend a systematic approach involving pulse palpation to detect irregularities, followed by ECG confirmation, for all patients in this age group presenting with an irregular pulse [49].

The rise of wearable devices, including smartwatches, rings, and bracelets, marks a significant advancement in healthcare technology. Their growing popularity stems from their unique combination of convenience, portability, and multifaceted capabilities. These devices provide flexibility by functioning either as standalone tools or in integration with mobile technologies like smartphones, thereby enhancing their capacity for continuous, real-time physiological monitoring and data acquisition [39]. Recent evidence underscores their clinical relevance, with 34% of individuals who received alerts for irregular pulse readings subsequently diagnosed with AF via ECG testing, and 84% of these alerts accurately identifying confirmed AF episodes [50].

Wang et al. compared the different wearable devices available on the market [51]. Zio patch is a single-lead ambulatory ECG patch that can monitor a patient’s ECG for up to 14 days. After the monitoring period, the patch is returned to the manufacturer. An algorithm is then used to detect cardiac arrest (CA) events and produce a report based on the ECG recording. Nuvant MCT (Mobile Cardiac Telemetry) patch (Corventis, Inc., San Jose, CA, USA) is a wearable, wireless arrhythmia detection system that enables arrhythmia detection in a user-friendly manner for both the patient and the physician, for up to 30 consecutive days. BodyGuardian patch ECG (Boston Scientific, Marlborough, MA, USA) with arrhythmia detection, respiratory rate, and activity monitoring. BardyDx CAM patch (Bardy Diagnostics, Inc., Bellevue, WA, USA) is designed to provide extended duration cardiac monitoring for people who are suspected of having cardiac arrhythmias. BioTel Heart patch gathers cardiac rhythm data from the sensor via Bluetooth, then sends these ECG data via a wireless connection. MediBioSense MBD HealthStream patch (Philips BioTelemetry, Malvern, PA, USA) is a real-time heart rate monitoring device. The ECG reports generated by the Vital patch (VitalConnect, San Jose, California, USA) wearable sensor offer complete medical analysis, available 24 hours a day, seven days a week. KardiaMobile patch is a single-lead ECG for arrhythmia detection and QRS elongation. In the case of the Apple Watch, the wristwatch is active whenever the watch is in use, lightweight, and user-friendly. SmartCardia INYU wristwatch (SmartCardia SA, Lausanne, Switzerland) 7/14-day patch provides seven-lead ECG in real-time and vital signs. PulseSmart is a smartphone-based app that analyzes pulse waveforms using the phone's camera and flashlight, with the user placing a finger on the camera. ECG Check (Cardiac Designs, Inc., Houston, Texas, USA) is a smartphone case that contains two metal electrodes connected to the phone, where a user places a single finger for 30 seconds for heart rhythm monitoring [51].

Several studies have demonstrated the potential of smartwatches that use AI-enabled algorithms for atrial fibrillation detection, offering convenient and accurate solutions for cardiovascular health management. Haverkamp et al. evaluated a smartphone-connected ECG to record a 30-second single-lead ECG in 144 individuals, demonstrating acceptable sensitivity and specificity for detecting pathological rhythms, including high accuracy for AF detection [52]. Similarly, a single lead ECG device with an integrated AF algorithm showed excellent diagnostic accuracy in primary care populations for AF and atrial flutter (AFL) [53]. In a case-control study with 150 AF patients and 150 controls, a three-minute mechanocardiography recording using a smartphone was compared to five-lead telemetry ECG, achieving 95.3% sensitivity and 96.0% specificity for AF detection [54]. Another algorithm using pulse wave signals from a smartphone camera achieved 95% sensitivity and specificity for AF [55]. The SAFETY (Screening for Atrial Fibrillation Using Economical and Accurate Technology) study assessed low-cost heart rate monitors (PH7 and BG2) in 418 patients, reporting >95% sensitivity and specificity for AF detection [56], with similar results after successful cardioversion [57]. Poh et al. proved that a deep convolutional neural network outperformed traditional methods for AF detection [58]. FibriCheck (Hasselt, Belgium), a smartphone-based PPG algorithm, showed 96% sensitivity and 97% specificity for AF detection in primary care [59]. Cardiio Rhythm (Cardiio, Cambridge, MA, USA) correctly identified 93.1% of AF cases and 90.1% of non-AF cases, achieving 93.1% sensitivity and 90.9% specificity, particularly effective in post-cardioversion patients [60]. A novel iPhone case with embedded electrodes for post-ablation monitoring achieved 100% sensitivity and 97% specificity for AF/AFL, making it a viable alternative to traditional monitors [61]. The AliveCor Kardia monitor with a parasternal lead demonstrated >95% sensitivity and specificity for AF detection [62]. The Kardia Mobile Cardiac Monitor (KMCM) showed 96.6% sensitivity and 94.1% specificity compared to 12-lead ECGs in detecting AF in 52 patients [63]. AliveCor (Mountain View, CA, USA) demonstrated accurate single-lead AF detection, offering a cost-effective, non-invasive option for routine nursing observations in stroke units [64]. The Ritmia (Heart Sentinel, Parma, Italy) app accurately identified AF in patients undergoing elective cardioversion, with 97% sensitivity, 95.6% specificity, and a kappa coefficient of 0.93, showing strong differentiation between AF and sinus rhythm [65] (Table 1).

**Table 1:** Characteristics of studies that evaluated smartwatches that use AI-enabled algorithms for atrial fibrillation detection. AF: atrial fibrillation, CAD: coronary artery disease, CHF: congestive heart failure, CV: cardioversion, DM: diabetes mellitus, ECG: electrocardiogram, EP: electrophysiologist, IM: internal medicine, IS: ischemic stroke, N/A: not applicable, PAC: premature atrial contraction, PPG: photoplethysmography, PVC: premature ventricular contraction, TIA: transient ischemic attack.

	Study	Country	Study design	Total number of participants	Mean age ± SD (years)	Participants	Inclusion of patients with history of AF	Total patients with history of AF (%)	AF detection methods	AF diagnosis period	Gold standard for AF diagnosis	Specificity (%)	Sensitivity (%)	Comorbidities (%)
1	Haverkamp et al. [52]	Norway	Prospective cohort	144	58	Voluntary citizens	Yes	11.7	N/A	30 seconds	12-lead ECG interpreted by cardiologists	94.0	100	N/A
2	Himmelreich et al. [53]	Netherlands	Retrospective cohort	214	64.1 ± 14.7	Participants aged > 18 years	Yes	10.7	Kardia (single-lead ECG)	30 seconds	12-lead ECG interpreted by cardiologists	97.9	87.0	Hypertension (40.7), DM (30.8), CAD (9.8), CHF (3.7), history of IS or TIA (6.1)
3	Jaakkola et al. [54]	Finland	Case-control	300	74.8	Hospitalized patients from IM and cardiology wards	Yes	50.0	Mechanocardiography	3 minutes	5-lead telemetry ECG interpreted by cardiologists	96.0	95.3	N/A
4	Krivoshei et al. [55]	Finland	Case-control	80	78 ± 7.5	Participants aged > 18 years	Yes	50.0	PPG	5 minutes	Recorded ECG	95.0	95.0	N/A
5	Lown et al. [56]	UK	Case-control (safety trial)	418	73.9 ± 6.1	Ambulatory patients aged > 65 years	Yes	19.6	AliveCora (single-lead ECG)	Not mentioned	12-lead ECG interpreted by cardiologists	98.81	87.8	N/A
6	McManus et al. [57]	USA	Retrospective cohort	121	66	Patients with history of AF, PVC, PAC	Yes	81.0	PPG	At least 2 minutes	12-lead ECG interpreted by cardiologists	93.5	97.0	Hypertension (74.4), DM (14.0), CAD (25.6), CHF (30.5), history of IS or TIA (12.4)
7	Poh et al. [58]	Hong Kong	Retrospective cohort	1013	68.4 ± 12.2	Patients without AF	Yes	None	PPG	51 seconds	12-lead ECG interpreted by cardiologists	99.0	95.2	Hypertension (90.4), DM (36.6), CAD (16.2), CHF (4.4), history of IS or TIA (10.5)
8	Proesmans et al. [59]	Belgium	Retrospective cohort	223	77 ± 8	Patients with known AF P	Yes	45.7	PPG	3 minutes	12-lead ECG interpreted by cardiologists	96.6	95.6	DM (20.2), CHF (28.7), history of IS or TIA (22.4)
9	Rozen et al. [60]	USA	Prospective cohort	98	67.7 ± 10.5	Patients aged > 18 years undergoing CV for AF	Yes	100	PPG	60 seconds	12-lead ECG interpreted by cardiologists	90.9	93.1	N/A
10	Selder et al. [42]	Netherlands	Prospective cohort	233	58.4 ± 14	Patients with recent diagnosis of arrhythmia	Yes	55	Single-lead ECG	30 seconds	Single-lead ECG interpreted by cardiologists	95.0	92.0	Hypertension (27.0), CAD (12.8), CHF (2.0)
11	Tarakji et al. [61]	USA	Prospective cohort	60	60 ± 12	Patients undergoing AF ablation	Yes	11.9	Single-lead ECG	30 seconds	Single-lead ECG interpreted by EPs	97.0	100	N/A
12	Wegner et al. [62]	Germany	Retrospective cohort	99	64 ± 15	Patients hospitalized to cardiac wards	Not mentioned	29.3	Single-lead ECG	Not mentioned	12-lead ECG interpreted by EPs	84.9	100	N/A
13	William et al. [63]	USA	Prospective cohort	52	68.1	Patients with AF receiving dofetilide or sotalol	Yes	35.4	Single-lead ECG	30 seconds	12-lead ECG interpreted by EPs	94.1	96.6	N/A
14	Tu et al. [64]	Hong Kong	Prospective cohort	217	70.3 ± 13.9	Patients hospitalized to cardiac wards	Not mentioned	34.6	PPG	60 seconds	12-lead ECG interpreted by cardiologists	95.8	94.7	Hypertension (59.9), DM (35.0) CHF (31.8), history of IS or TIA (18.9)
15	Reveberi et al. [65]	Italy	Prospective cohort	100	66.2 ± 10.7	Patients aged > 18 years undergoing CV for AF	Yes	91.6	Wearable chest strap (R-R interval analysis)	10 minutes before and after CV	12-lead ECG interpreted by cardiologists	95.6	97.0	N/A

Data privacy and ethical concerns

The lack of clear legal and regulatory frameworks for data rights may be attributed to the recent diffusion of AI in specific areas of healthcare, the emergence and untimely development of new devices, and inconclusive evidence, which leads to the introduction of products with unknown safety and efficacy. These devices considered low-risk, despite receiving FDA clearance [66], do not undergo a formal FDA approval process, which requires extensive testing. This opens a window of opportunity to bypass rigorous scientific validation, potentially putting the user at risk [67].

Although these devices promote patient empowerment, autonomy can be violated, as smartwatches currently do not provide information equivalent to formal medical counseling. Therefore, decisions are often ill-informed, and the risks or implications of screening and false-positive or false-negative results are not addressed. It is believed that understanding the fairness, accountability, transparency, and explainability of AI systems is vital to increasing user trust [68]. One of the problems, often overlooked, is that most AI developers are men, and the male thought model may place women at a disadvantage. The involvement of private companies with singular regulations and objectives raises concerns about data security.

Another important point is the growing emergence of evidence regarding the significant disparity in cardiovascular outcomes between different populations, influenced by factors such as socioeconomic status and ethnicity [69, 70]. Low- and middle-income countries account for approximately 80% of the global burden of cardiovascular diseases (CVD) [71], and the fact that most studies evaluating the effect and safety of these devices were developed in high-income countries raises ethical and safety concerns that deserve particular attention.

Despite the higher incidence, prevalence, and complications of AF in the elderly, studies show that smartwatches are predominantly used by young individuals, mostly white, with higher socioeconomic status. Another important issue that deserves particular attention is the fact that all trials examining the use of smartwatches for AF detection have been conducted in high-income countries. Although women with AF have a higher mortality risk compared to men, only 29% of smartwatch users are women [72, 73].

These ethical challenges intersect with the issue of health equity. As such, these aspects of using smartwatches in screening patients with AF may exacerbate disparities in healthcare. However, data sharing and the development of new trials aimed at identifying patient groups who may benefit most from smartwatch-based screening are an important part of making health equity possible [74].

Challenges and future perspectives

When a smartwatch detects a possible case of AF, the key question is how healthcare providers should proceed [66]. In existing clinical studies, participants were advised to consult a healthcare provider [14,39,75], who would then confirm the presence of AF through a standard ECG test. Mobile health technologies, such as mHealth apps and wearable activity monitors, are rapidly advancing in the detection of AF, according to the 2020 ESC (European Society of Cardiology) Guidelines [9]. However, caution is warranted regarding the clinical use of these technologies, as many have not yet undergone rigorous clinical validation. Several studies have explored AF detection through smartwatches, revealing promising new possibilities for identifying populations at higher risk. Moreover, the application of machine learning and artificial intelligence could enable the identification of individuals who have had previous AF episodes by analyzing sinus rhythm ECG recordings, which could represent a major advancement in AF detection [9].

The management of data from smartwatches presents a significant challenge. As the use of wearable devices capable of detecting AF continues to grow exponentially, from 325 million devices in 2016 to 1.1 billion devices in 2022 globally [76], there is an increasing need to consider the commercial factors involved. The development of these devices is driven by numerous technical and scientific collaborations, yet their widespread integration into medical services remains a challenge. The rapid pace of their development has outpaced the necessary evaluation of their reliability and effectiveness in clinical settings, underscoring the need for more comprehensive assessments.

Currently, the FDA does not classify smartwatches as medical devices; instead, they are regarded as wellness tools, which are subject to expedited approval under the Digital Health Software Pre-Certification Program (Pre-Cert) [77]. On one hand, the introduction of smartwatches for AF diagnosis could result in a significant burden on the healthcare system, as the routine use of these devices may lead to increased demand for services, placing additional stress on healthcare providers [78,79].

## Conclusions

Smartwatches offer a non-invasive, easy-to-use tool for the early detection of AF, especially in high-risk asymptomatic individuals. However, further research, including large-scale trials, is needed to validate their clinical impact, optimize AI algorithms, and address issues such as identifying suitable populations and ensuring the protection of health data.
